# Effectiveness of medical hypnosis for pain reduction and faster wound healing in pediatric acute burn injury: study protocol for a randomized controlled trial

**DOI:** 10.1186/s13063-016-1346-9

**Published:** 2016-04-29

**Authors:** Stephen J. Chester, Kellie Stockton, Alexandra De Young, Belinda Kipping, Zephanie Tyack, Bronwyn Griffin, Ralph L. Chester, Roy M. Kimble

**Affiliations:** Centre for Children’s Burns and Trauma Research, Level 7, Centre for Children’s Health Research, University of Queensland, 62 Graham Street, South Brisbane, QLD 4101 Australia; School of Medicine, Mayne Medical School, The University of Queensland, 288 Herston Road, Herston Brisbane, QLD 4006 Australia; Ochsner Clinical School, Ochsner Hospital, 1514 Jefferson Highway, New Orleans, LA 70121 USA; Horizon Behavioral Health, 2241 Langhorne Road, Lynchburg, VA 24501 USA

**Keywords:** Burns, Child, Hypnosis, Hypnotherapy, Pain, Stress, Anxiety, Posttraumatic stress disorder, Salivary α-amylase, Randomized controlled trial

## Abstract

**Background:**

Burns and the associated wound care procedures can be extremely painful and anxiety-provoking for children. Burn injured children and adolescents are therefore at greater risk of experiencing a range of psychological reactions, in particular posttraumatic stress disorder, which can persist for months to years after the injury. Non-pharmacological intervention is critical for comprehensive pain and anxiety management and is used alongside pharmacological analgesia and anxiolysis. However, effective non-pharmacological pain and anxiety management during pediatric burn procedures is an area still needing improvement. Medical hypnosis has received support as a technique for effectively decreasing pain and anxiety levels in adults undergoing burn wound care and in children during a variety of painful medical procedures (e.g., bone marrow aspirations, lumbar punctures, voiding cystourethrograms, and post-surgical pain). Pain reduction during burn wound care procedures is linked with improved wound healing rates. To date, no randomized controlled trials have investigated the use of medical hypnosis in pediatric burn populations. Therefore this study aims to determine if medical hypnosis decreases pain, anxiety, and biological stress markers during wound care procedures; improves wound healing times; and decreases rates of traumatic stress reactions in pediatric burn patients.

**Methods/Design:**

This is a single-center, superiority, parallel-group, prospective randomized controlled trial. Children (4 to 16 years, inclusive) with acute burn injuries presenting for their first dressing application or change are randomly assigned to either the (1) intervention group (medical hypnosis) or (2) control group (standard care). A minimum of 33 participants are recruited for each treatment group. Repeated measures of pain, anxiety, stress, and wound healing are taken at every dressing change until ≥95 % wound re-epithelialization. Further data collection assesses impact on posttraumatic stress symptomatology, speed of wound healing, and parent perception of how easy the dressing change is for their child.

**Discussion:**

Study results will elucidate whether the disease process can be changed by using medical hypnosis with children to decrease pain, anxiety, and stress in the context of acute burn wounds.

**Trial registration:**

Australian New Zealand Clinical Trials Registry ACTRN12615000419561

## Background

### Global pediatric burns burden

Worldwide numbers of annual pediatric hospital admissions for burn treatment vary geographically from a rate of 4.4/100,000 total population in America (North, Central, and South) to 10.8/100,000 total population in Africa [1]. Every day in the USA approximately 300 children aged 0 to 19 years receive treatment in emergency departments for burn injuries and two children die from burns [2]. In Australia, approximately 40.1/100,000 children aged 0 to 14 years are hospitalized annually due to burns and scalds [3]. While pediatric burn mortality rates are decreasing worldwide, the morbidity attributed to burns due to factors such as pain, psychological distress, and physical impairment is increasing [4]. Thus, it is important to identify the most effective interventions to reduce the burden of burns.

### Pediatric burn pain

In modern medicine, pain has moved beyond concern as a mere disease symptom and is now considered a basic human rights issue [5]. Burns and the associated wound care procedures (e.g., wound cleaning, debridement, and dressing) can be painful for children [6]. After the initial burn is sustained, procedural pain remains both the most intense and undertreated type of pain despite continual advancements in burn wound care [6]. Furthermore, many patients indicate that wound care procedures are as painful as the original burn insult (and occasionally *more* painful), provoking intense anticipatory anxiety [7].

### Psychological distress

In addition to being painful, burn injuries can result in severe psychological distress [8]. Burn injury leads to an increased risk of children developing a range of major mental illnesses, in particular posttraumatic stress disorder (PTSD) [9, 10]. Several clinical studies have identified traumatic stress reactions in preschool children in the first year post-burn, ranging between 25 and 30 % in the acute phase to approximately 10 % one year after the burn [8]. Acute stress is prevalent in approximately one third of school-aged children post-burn, and qualitative, cross-sectional studies have identified current PTSD in 10–20 % of the children and young adults many years post-burn [8]. A prospective observational study in our burns center with 130 burn injured children found that 35 % were diagnosed with at least one psychological disorder, with a high comorbidity rate of PTSD [11]. Furthermore, prior research indicates that a clinically significant relationship may exist between symptoms of psychological distress and burn pain, each of which can exacerbate the other [12]. Unaddressed fears and anxiety contribute to noncompliance and can complicate pain management and healing [13]. Thus, treatment options that alleviate pain and distress must be offered to patients for optimal care [14].

### Physiologic effects of pain and stress on wound healing

It is worth studying endogenous pain mediators in children, not only for compassionate reasons of pain control, but also in the context of their direct physiologic effects on wound healing [15]. Widgerow theorizes that greatly increased pain mediator release in the context of burns may result in nociceptors becoming overly sensitized, increased inflammatory cellular and extracellular matrix alterations, and possibly increased hypertrophic scarring risk [15]. Hypertrophic scarring, a common thermal injury complication, is manifested by excessive collagen deposition in the healing wound bed [16]. Therefore, aside from modulating a child’s subjective (i.e., central) interpretation of pain, decreasing acute burn pain can potentially promote faster wound healing and improve long-term scar outcomes by downregulating local pain mediator release [15].

In humans the release of glucocorticoids (e.g., cortisol) and catecholamines (e.g., epinephrine and norepinephrine) elicits the classic “fight, flight, or freeze” response when psychological stress is experienced [4]. Herndon et al. have demonstrated increased levels of epinephrine and norepinephrine sustained for up to 35 weeks in children post-burn, providing evidence of the magnitude and duration of the catecholamine surge encountered in this population [17, 18]. These physiologic effects in response to stress are important, as a number of studies and meta-analyses have implicated psychological stress in significantly delaying cutaneous wound healing [19–21]. Attention to and relief of pain, anxiety, and stress for burned children is therefore a high clinical priority [13].

### Pharmacological and non-pharmacological pain and anxiety management

To prepare pediatric patients for burn wound care procedures, a pharmacological protocol is usually employed for analgesia and anxiolysis [14]. A variety of non-pharmacological techniques are also used adjunctively with pharmacological methods for pain and anxiety control. Distraction and preparation techniques and devices have demonstrated benefit in pediatric burn patients [6, 22–24]. Importantly, pain reduction during burn wound care procedures has been linked with clinically significant improvement in wound healing (i.e., re-epithelialization) rates [6, 25, 26].

### Medical hypnosis

Medical hypnosis helps patients focus their attention to lessen pain and anxiety and enhances patients’ acceptance of clinicians’ positive suggestions to change or reframe their perceptions, sensations, thoughts, and behaviors [27]. Hypnotherapeutic techniques have decreased pain and anxiety in the short term and decreased psychological distress over the long term, thereby optimizing patient outcomes and complementing existing treatment modalities [28]. Medical hypnosis can also empower pediatric patients to assist themselves at will beyond the presence of the therapist by teaching them self-hypnosis, which engenders self-mastery and active participation in their own treatment [29].

In children, clinical hypnotherapy techniques including hypnoanalgesia and hypnoanesthesia (hypnotically induced analgesia and anesthesia, respectively) have alleviated acute pain associated with a number of painful pediatric medical procedures [30]. There is a growing body of evidence supporting medical hypnosis’ ability to reduce pain and anxiety associated with venipuncture, bone marrow aspiration, and lumbar puncture in children [31]. A recent review of studies on the effectiveness of medical hypnosis for reducing procedure-related pain in children and adolescents less than 19 years old consistently found that hypnosis was more effective than control conditions in alleviating discomfort associated with bone marrow aspirations, lumbar punctures, voiding cystourethrograms, the Nuss procedure (surgery to correct congenital deformities of pectus excavatum), and post-surgical pain [32]. Furthermore, all controlled studies included in the review found the effectiveness of hypnosis to be either equal or superior to that of distraction [32].

In adults, medical hypnosis during burn wound debridement has resulted in clinically significant pain and anxiety reduction [7, 33–35]. Additionally, adult hypnotherapy has repeatedly demonstrated effectiveness in treating pain and psychological distress incurred by a number of classically uncomfortable procedures including tooth extraction [36], bone marrow aspiration [37], and colonoscopy [38].

Prior research has shown that children over the age of three respond to medical hypnosis [30, 39] and that hypnotic responsivity (historically known as “hypnotizability”) is generally greater in children than in adults [29, 39]. It has been proposed that hypnotherapy may be the preferred non-pharmacological intervention for young children, given how easily and fluidly they enter trance-like states (e.g., playing with imaginary “friends”) [29, 40]. The hypnotic induction of an altered state of consciousness in children is characterized by narrowed attention, absorption in trance phenomena, and some degree of detachment from the surrounding external environment. Hypnotic intervention provides a context in which children can exercise their curiosity while producing a novel experience which increases their mastery over physical and mental response patterns. In the younger child, play is a natural form of expression and problem-solving which uses altered levels of consciousness to vivify the child’s experience. Thus, the normally developing child has a naturally large repertoire of imaginative experience to draw on when provided with a hypnotherapeutic intervention [41]. Despite children’s generally greater responsivity to hypnotherapy compared to that of adults, to our knowledge no randomized controlled trial (RCT) has examined the effectiveness of medical hypnosis for decreasing pain intensity, healing times, procedural anxiety, and rates of traumatic stress reactions in pediatric burn patients. Together these findings, in addition to the ease of application, lack of adverse side effects, and cost-effectiveness of medical hypnosis with children [42], provide a strong rationale for implementing this study.

### Objectives

The primary aims of this study are to investigate whether medical hypnosis affects pain intensity and the rate of burn wound healing (i.e., re-epithelialization) in acutely burned children. The secondary aims are to investigate if medical hypnosis affects procedural anxiety, biological stress markers (salivary cortisol and salivary α-amylase), and the rate of PTSD symptom development. We hypothesize that use of medical hypnosis for pediatric patients with acute burns will decrease pain intensity, procedural anxiety, and biological stress markers during wound care procedures; improve wound healing times; and decrease rates of traumatic stress reactions compared to a standard care control group.

## Methods/Design

### Protocol and registration

This study has received ethical approval from the Queensland Children’s Health Services (Lady Cilento Children’s Hospital) Human Research Ethics Committee (approval number: HREC/15/QRCH/32) and the University of Queensland Ethics Committee (approval number: 2015000456). The study methodology was documented in a protocol and registered prior to starting recruitment (Australian New Zealand Clinical Trials Registry, ID: ACTRN12615000419561). This is version 1 of the study protocol completed on 10 September 2015. Methods have been documented in accordance with the Consolidated Standards of Reporting Trials (CONSORT 2010) [43] and Standard Protocol Items: Recommendations for Interventional Trials (SPIRIT 2013) [44] statements. The intervention has been described using the Template for Intervention Description and Replication (TIDieR 2014) guidelines [45].

### Design and setting

**Fig. 1:**
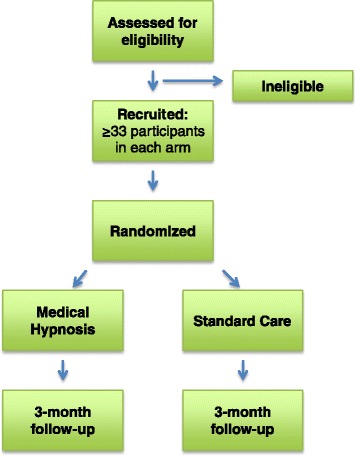
Flow diagram of the trial

This study is a single-center, superiority, parallel-group, prospective randomized controlled trial (see Fig. 1). Eligible participants are randomized to receive either (1) medical hypnosis (intervention group) or (2) standard care (control group).

Participants are recruited from the Pegg Leditschke Paediatric Burns Centre (PLPBC) at Lady Cilento Children’s Hospital (LCCH), Brisbane, Australia (AUS). The PLPBC is the major specialist tertiary burns center for Queensland and Northern New South Wales, Australia. The center’s clinical multidisciplinary team treats approximately 800 new burn patients per year.

### Eligibility criteria for participants

Eligible participants are children aged between 4 and 16 years (inclusive) who meet the inclusion criteria of (1) an acute burn of any depth (excluding erythema only) and (2) presentation to the PLPBC for treatment (inpatient or outpatient). Children are excluded from the study if they are non-English speaking; cognitively impaired; under the care or investigation of the Department of Communities, Child Safety, and Disability Services; on ventilator support; or if they have initial burn wound care procedures carried out in the operating room under general anesthesia.

Patients of the PLPBC are identified by the center’s research manager, and participants’ eligibility is assessed by the nursing staff. All eligible patients presenting to the PLPBC are approached and invited to participate in this RCT by a clinical research team member not involved in their primary care. Parent (the term *parent* includes *legal guardian*) informed consent is obtained and recorded. Child consent is obtained for all children able to read and write. Verbal assent is obtained for all other eligible children.

### Interventions

Recruited participants are randomized into either (1) the intervention group: medical hypnosis or (2) the control group: standard care immediately prior to their first wound care procedure (i.e., dressing change or application, which may involve cleaning the wound) at the PLPBC:

1. Intervention group
  Medical hypnosis
    Medical hypnotherapy is used with this group after gathering baseline data. Hypnotic induction (i.e., the method for guiding the participant into a hypnotic trance) starts before nursing staff begin the burn wound care procedure and is maintained throughout the procedure. The hypnotic induction is adjusted for the participant’s age, understanding, and communicative capacity as per the clinician’s judgment. After the first hypnotherapy session (concurrent with the participant’s first wound care procedure), a maximum of four additional hypnotherapy sessions are provided during subsequent visits to the PLPBC for wound care procedures. The clinical judgment of the treating hypnotherapist in consultation with the burns nurses and pediatric surgeons is used to determine the total number of hypnotherapy sessions. Aside from the hypnotherapy, all other treatment is administered according to established standard care. Standard care at PLPBC includes administering oxycodone, with the dosage determined by body weight, 0.1 mg/kg orally (Mundipharma Pty. Ltd., Sydney, NSW 2000, Australia); and paracetamol, 150 mg/kg orally (Sanofi-Aventis AUS Pty. Ltd., Macquarie Park, NSW 2113, Australia).
  Essential elements of medical hypnosis
    The medical hypnosis intervention follows five stages: establishing rapport and creating a setting of positive expectancy; slowing breathing and enhancing relaxation; providing suggestions for deepening relaxation and absorption in the hypnotic state; direct hypnotic suggestions for hypnotically induced analgesia, anesthesia, anxiolysis, and rapid healing; and alerting (i.e., bringing the participant out of the hypnotic trance) [46].
  Materials
    The medical hypnosis intervention designed for the treatment purposes of this trial has been documented in a manual and can be obtained from the study’s principal investigator on request.
  Procedures
    This study uses two hypnotic inductions: the Favorite Stories induction [47] (for children less than 7 years old) and the Favorite Place induction [29] (for children 7 years old and older). Once trance is achieved, suggestions for hypnoanalgesia or hypnoanesthesia are given using the Switches method [29, 48] or the Glove Anesthesia method [29] (sometimes referred to as the Magic Glove in the relevant literature). Suggestions for rapid wound healing are also given, and self-hypnosis is taught to the child [29].
  Provider
    The medical hypnosis provider in this study is a medical student and Research Higher Degree candidate who received group training by the American Society of Clinical Hypnosis (ASCH) and individual face-to-face training with Dr. Dabney M. Ewin, M.D., FACS, ABMH, Professor of Surgery and Psychiatry at Tulane University, New Orleans, USA and former president of both the ASCH and the American Board of Medical Hypnosis.
    All medical hypnosis is delivered face to face in a clinical consulting room within the PLPBC, LCCH. A minimum of one session and maximum of five sessions of medical hypnosis are provided for each participant in the intervention group, concurrent with their scheduled wound care procedures. Each session lasts for the duration of the participant’s wound care procedure.
    Although the medical hypnosis manual is adhered to as closely as possible, the intervention is tailored to the participant based on their developmental and chronological age, capacity to focus, distress related to the total burn area and depth, acuity and nature of the burn event, and location of the burn. This adherence allows replication of hypnotherapy procedures used to the extent permitted given that the children and adolescents who participate have varied developmental and chronological ages, presentations, and levels of capacity for focus, as well as distress related to total burn area and depth, acuity and nature of the burn event, and different specific anatomy involved.
2. Control group
  Standard care
    Standard procedural distraction is available to this group, including music, toys, the Ditto™ (Diversionary Therapy Technologies, QLD, Australia) [6] and other electronic devices (e.g., TV, hand-held games, portable DVD players), books, and parental presence. The consulting hypnotherapist is positioned in the procedure room with the participant but does not provide hypnosis to ensure that the only difference is the use of medical hypnosis. The hypnotherapist does not interact with participants in the control group to avoid potentially using hypnotic techniques and is only present to help record outcome measures associated with wound care procedures.

### Outcomes

Previously validated scientific measures are used for all outcomes. The duration of each wound care procedure is timed. Prior to the application of new dressings at the first dressing change, the burn depth is calculated by measuring blood perfusion at the burn site using a Moor LDI2-BI2 Laser Doppler Imager (Moor Instruments Limited, Devon, UK). Data is collected at every dressing change until ≥95 % re-epithelialization occurs, and the total number of dressing changes is recorded.

#### Primary outcome measures

The two primary outcome measures are pain intensity and wound healing (i.e., re-epithelialization time). Pain measurements are taken several times for each participant. The first time point (baseline) is immediately before premedication prior to removal of the wound dressing in clinic. The second time point is immediately after the new dressing is in place; the worst pain is assessed reflecting the maximal pain intensity experienced by the participant during the procedure (retrospective). For the third time point, a final pain assessment occurs to gauge pain intensity immediately after the new dressings are in place and hypnotherapy has ceased. No pain measurements are taken during hypnosis. Assessing pain intensity in this manner effectively gives three time points relative to the procedure: before, during, and after. These measurements take place at each subsequent dressing change until ≥95 % wound re-epithelialization.

#### Pain

A range of pain scales are utilized to measure pain intensity (assessed by child, nurse, or parent). The Faces Pain Scale-Revised (FPS-R) [49] is used to assess the child’s self-report of pain and will be the primary outcome measure. Convergent validity of the FPS-R is supported by a strong positive correlation (*r* = 0.93, *p* < 0.001, *N* = 76) with a visual analog scale (VAS) pain intensity measure in children aged 5–12 years and by strong positive correlations with the VAS (*r* = 0.92, *p* < 0.001, *N* = 45) and the color analog scale (*r* = 0.84, *p* < 0.001, *N* = 45) in a clinical sample of pediatric inpatients aged 4–12 years [49]. Even among the youngest patients sampled (four-year-olds), there is evidence of usage of the FPS-R and analog scales in a consistent and reliable manner [49]. Nurses report a behavioral/observational rating on the Face, Legs, Arms, Cry, Consolability (FLACC) scale [50, 51]. The FLACC scale has been validated for use in settings such as postoperative pain [50]. Despite a recent systematic review rescinding recommendation of the FLACC scale for procedural pain assessment [51], we have chosen this scale in the absence of an acceptable alternative due to its extensive use in prior clinical trials examining procedural pain. Parents also rate their child’s pain using an 11-point (0 to 10) numeric rating scale (NRS) [52]. Pain scores reported verbally by the parent (NRS) and child (FPS-R) are documented by the primary investigator. Nurses document the FLACC pain scores.

#### Wound healing

Re-epithelialization is defined as ≥95 % wound healing and no further wound dressings required. Scabs or crusts are defined as unhealed areas [53]. For the purpose of the main analysis, this outcome measure will be assessed using the percentage re-epithelialization assessed from 3D digital photography by an independent surgeon and nurse blinded to study treatment group. The percentage re-epithelialization reported by the independent surgeon will be used in the main analysis if appropriate. The outcome will also be measured from 3D digital photography by the investigator (SJC), and the time to wound healing recorded in the medical records will also be used for comparative purposes. Wound photographs are taken at each dressing change using 3D LifeViz System™ (Quantificare, Sophia Antipolis, France) [54]. The independent blinded surgeon and nurse will mark out the wound edges after the photographs are taken, along with any unhealed areas. Surface area computer mapping will then be used to determine percentage re-epithelialization [53, 54].

#### Secondary outcome measures

Secondary outcome measures collected at baseline include a self-reported procedural anxiety measure, a saliva sample (for measurement of stress biomarkers: salivary cortisol and salivary α-amylase), and heart rate (HR). Baseline measurements are taken before nurses administer pharmacological analgesia according to PLPBC standard practice.

#### Procedural anxiety

The visual analog scale for anxiety (VAS-A) [55, 56] will be used to measure procedural anxiety in children. Self-reported anxiety measures will only be administered to children 8 years old and above. For participants younger than 8 years, the parent will be asked to assess their child’s anxiety using the same scale. The anxiety measurement is obtained prior to premedication and immediately after new dressing application.

#### PTSD

PTSD severity three months following injury will be assessed using the Child PTSD Symptom Scale (CPSS) [57] for children aged 7 years or older. The CPSS is designed to assess PTSD diagnosis and symptom severity in children ages 8 to 18 who have experienced a single-incident traumatic event [57]. Total symptom score and the three symptom clusters of the CPSS demonstrate high internal consistency (α = 0.89 for total score) [57]. The percentage agreement between PTSD diagnoses at two separate time points was 84 %, indicative of moderately high reliability [57]. Test-retest reliability of the total CPSS score is acceptable (κ = 0.84) [57]. Convergent validity of the CPSS has been supported (Pearson’s *r* = 0.80, *p* < 0.001) when measured against the Child PTSD Reaction Index (CPTSD-RI) [57].

The Young Child PTSD Checklist (YCPC) [58] will be used for children younger than seven years old. Face validity of the YCPC items is excellent based on a series of studies by Scheeringa et al. that used these items in an interview format and formed the basis for the new DSM-5 disorder titled “Posttraumatic stress disorder for children six years and younger” [59–62]. The test-retest reliability (intraclass correlation coefficient = 0.87) [63] was acceptable, and the predictive validity [64] of PTSD symptoms using an interview format has been supported.

#### Parent satisfaction

Ease of the child’s wound care procedure as assessed by the parent is a secondary outcome measure. At the conclusion of every dressing change, a parent rates how easy they believe the wound care procedure was for their child on a 5-point Likert scale (from “not at all easy” to “extremely easy”) with higher values indicating greater satisfaction.

#### Biochemical stress markers

Salivary cortisol, representing hypothalamic-pituitary-adrenal axis activity, and salivary α-amylase (a proxy for norepinephrine indicative of sympathetic adrenomedullary system activity) are measured as biomarkers of stress associated with wound care procedures [65]. The participant places a Salivette™ (Sarstedt Australia Pty., Ltd. Mawson Lakes, SA, Australia) under their tongue for 2 minutes for saliva collection at these times: (1) immediately before premedication prior to removal of wound dressing in clinic, (2) immediately after the new dressings are in place, and (3) three months post-injury to obtain a baseline. The parent completes a saliva collection survey which records variables pertinent to salivary analysis: collection time, time participant last woke up, time participant last brushed teeth, any medication given, any food/drink/gum during the previous hour, time participant last had any caffeine, and pertinent smoking or tobacco history.

The date, time, and volume of saliva collection are recorded in the laboratory and samples are refrigerated at 4 °C and processed within 7 days. Samples are spun in a centrifuge at 1400 × *g* at room temperature for 10 minutes and the saliva frozen at −80 °C until analysis. Salivary cortisol and α-amylase will be quantified using ELISA kits (Stratech Scientific, Avalon, NSW, Australia) with saliva samples analyzed in triplicate. Heart rate will also be recorded as a physiologic measure of pain and distress at time points (1) and (2) for each dressing change.

#### Hypnotic responsivity

The Stanford Hypnotic Clinical Scale for Children (SHCS-C) [66] will be used to assess and record hypnotic responsivity for participants in the intervention group as recommended by prior methodological reviewers of relevant literature [32]. This assessment will only be conducted within the intervention group to ensure that control participants remain naïve to hypnotherapy. Normative data for hypnotic responsivity are available for children aged 3–16 years. The SHCS-C correlated 0.67 with a slightly modified version of the Stanford Hypnotic Susceptibility Scale, Form A for pediatric use [66], supporting concurrent validity.

### Demographic and clinical information

Participant demographics and medical history are recorded from the caregiver and hospital chart: mechanism and site of injury, estimated percentage total body surface area (TBSA) of burn, burn depth, any first aid treatment applied, and medication given. TBSA is determined by a consultant surgeon using the Lund and Browder method [67].

### Participant timeline

Participants are enrolled after presenting to the PLPBC and after their study eligibility has been assessed (Fig. 1). Regardless of which trial arm they are randomized into, all participants receive treatment during their scheduled appointment times for burn wound care at the PLPBC. Primary outcome data is collected concurrently with participants’ scheduled wound care procedures until the burn wound is ≥95 % re-epithelialized. The endpoint for secondary outcome data collection is 3 months post-burn. No extra participant visits to the PLPBC are required for the sole purpose of data collection.

### Sample size

A sample size estimate was derived from the primary outcomes: days to re-epithelialization and pain. Based on re-epithelialization within 15 (SD = 4) days and a minimum clinically important difference (MCID) of 3 days [26], the sample size required was estimated as 29 per group, using 80 % power and an α of 0.05. Allowing for 10 % loss to follow-up, a total of 66 participants will be required. Additionally, this sample size is adequate to show an MCID of 2 (SD 2.5) in the pain outcome measures [26]. Recruitment will continue until at least 33 participants in each arm have been obtained with complete data for the primary outcomes.

### Randomization

A computerized random number generator is used to randomize study participants. Simple randomization is overseen by staff not involved with the study. Third-party concealment of group allocation occurs by using a numbered series of opaque, sealed envelopes prepared in advance. The primary researcher is then told to which group the participant is allocated.

### Blinding

Medical hypnosis provided throughout a procedure cannot be masked. This study’s nature prevents full blinding, but certain outcome measures are blinded. The re-epithelialization assessors are blinded, as these measurements take place using 3D digital photographs. Trial group allocation remains unknown to this assessor. If discrepancy arises between the two re-epithelialization assessors, the assessment of the blinded assessor is taken as definitive to reduce potential performance bias. Burn depth and salivary analysis are also blinded measures, as data for these variables are provided to the investigators in a non-identifiable format and as the assessor of these variables is blinded to trial group allocation.

### Discontinuation

Study participants can withdraw from the trial at any time. The number of adverse events will be documented, and any adverse event will be described in detail for both treatment groups. Current relevant literature has not reported any serious harmful effects associated with indicated pediatric medical hypnosis or hypnotherapy.

### Data analysis

Data will be analyzed using SPSS 23 (IBM Corporation, Armonk, NY, USA). Descriptive statistics such as the mean and standard deviation, median and interquartile range, and confidence intervals will be used to report the sample demographics (i.e., age, gender, and mechanism of injury) and to summarize outcome measures, as appropriate. Between-group comparisons will be conducted for potential confounding variables for the primary outcomes. Potential confounding variables affecting wound healing that will be examined include burn depth, days taken to present to the PLPBC, ethnicity, mechanism of injury, percent TBSA, age, and gender [25]. Potential confounding variables affecting pain intensity that will be examined include age, the presence/absence of skin grafting, state anxiety (determined by VAS-A), pharmacologic analgesia given immediately before or during wound care procedures, percent TBSA, and days to re-epithelialization [26, 68]. If significant between-group differences are present for potential confounding variables, those variables will be controlled for in the primary analyses. Between-group differences will be investigated using univariate parametric or non-parametric analyses as applicable (e.g., linear regression, Student’s *t* test, or Mann-Whitney *U* test for continuous data and the chi-squared test or Fisher’s exact test for categorical data). All data will be analyzed on the intention-to-treat (ITT) principle as the primary approach. However, a sensitivity analysis will be conducted for data collected as per protocol. A repeated measures analysis will be undertaken using generalized estimating equations (GEEs) [69] including the main effect of treatment group and time on the primary pain and healing outcomes, as well as on the secondary outcomes of procedural anxiety, PTSD, parent satisfaction, and biochemical stress markers.

Analyses will be conducted with data stratified for burn depth (superficial partial-thickness/deep partial-thickness/full-thickness) and participant age (e.g., <8 years/≥8 years, with age strata based on age-group validity of the VAS-A) [69]. Differences in hypnotic responsivity between the intervention group and a normative comparison group will be analyzed using z-scores or using equivalent non-parametric tests such as the Mann-Whitney *U* test where applicable. Differences in re-epithelialization by the independent blinded raters (surgeon and nurse) and the investigator (SJC) will be examined using reliability coefficients and measures of agreement (e.g., percentages of exact agreement). Differences between measures of pain (e.g., observer report by parents versus child self-report) will be examined using correlational analyses where appropriate.

The influence of demographic and clinical factors, and primary and secondary outcomes not included as dependent variables, on primary and secondary outcomes will be examined using regression models and GEE models. Post hoc adjustment for multiple comparisons will be conducted using the Šidák correction [70] where appropriate. Statistical significance will be set at *p* < 0.05.

### Data storage

Data are protected in locked filing cabinets within the secure area of the Centre for Children’s Health Research, University of Queensland. Data are entered into a spreadsheet using Excel. Any incomplete data are coded as unknown, missing, or not applicable. The data set will be cleaned, checked, and then locked for analysis. Upon trial completion, data will be stored for 15 years as stipulated by the Queensland Children’s Health Services (LCCH) Human Research Ethics Committee.

### Dissemination

Outcomes will be published in a peer-reviewed medical journal (publication target *Burns*) and will also be reported at relevant conferences.

## Discussion

To our knowledge, this is the first RCT in the field of pediatric burn care investigating the impact of medical hypnosis on pain intensity, wound healing, procedural anxiety, biological stress markers, and PTSD development associated with wound care procedures. The neural mechanisms responsible for the antinociceptive effects of hypnosis have been rigorously studied by Rainville et al. [71, 72] and Faymonville et al. [73–78] by measuring regional cerebral blood flow (rCBF) using positron emission tomography. They independently reached the same conclusions: (1) Both the intensity (sensory component) and unpleasantness (affective component) of noxious stimuli are reduced during the hypnotic state, and (2) hypnotic pain modulation is chiefly facilitated by the anterior cingulate cortex [73]. Functional brain imaging studies like these not only help prove the existence of a hypnotic state but also validate the therapeutic effects of hypnotherapy in the medical setting [78].

As noted by early nineteenth-century physicians and surgeons going at least as far back as Dr. James Braid (1795–1860), children and adolescents are especially “sensitive” to hypnotic techniques and responsive to hypnotherapeutic strategies for patient care [29]. In terms of clinical benefits, hypnotherapy can aid in decreasing at least two facets of the complex, multidimensional phenomenon of pain: the sensory component (tied to pain intensity) and the affective component (tied to the emotional experience of pain) [79]. In addition to hypnotic suggestions for analgesia and anxiolysis, the hypnotherapist is uniquely able to give posthypnotic suggestions prior to ending the pediatric patient’s trance.

Barber explains that posthypnotic suggestions are capable of causing dissociation of noxious perceptions, aiding in reducing the sensory and affective components of pain [80]. Successive hypnotherapy sessions have a cumulative effect, resulting in neuroplastic changes. Neural reorganization is thought to occur, such that pain responses are replaced by non-painful responses devoid of suffering that develop in response to originally noxious stimuli [80]. Barber also identifies the hypnotic effect as being enhanced by the clinical relationship and the establishment of good rapport [80].

Since one classic element of the therapeutic hypnotic state is a feeling of relaxation (i.e., increased parasympathetic tone), anxiety reduction goes hand in hand with clinical hypnosis. A randomized trial (*n* = 50, age 2–11 years) conducted by Calipel et al. compared the efficacy of hypnosis to reduce anxiety and perioperative behavioral disorders versus midazolam premedication in children [81]. The number of anxious children was significantly less during anesthetic induction in the hypnosis group (39 % versus 68 %, *p* < 0.05) [81]. Hypnosis reduced the frequency of postoperative behavioral disorders by about half on day 1 and day 7 relative to midazolam [81]. These findings suggest that hypnosis is more efficient at reducing preoperative anxiety in children compared to midazolam, with the added benefit of reducing behavioral disorders in the first two postoperative weeks.

Given the ease with which even preschool-aged children [48] use and learn simple self-hypnosis techniques for pain and anxiety management, and considering that a child’s successful use of self-hypnosis facilitates a sense of self-mastery and empowerment, this RCT seeks to validate medical hypnosis as a useful tool and opportunity to help manage or prevent the pain, distress, and negative psychological sequelae commonly associated with pediatric burns and wound care procedures.

### Significance of the study

Pediatric burn pain, anxiety, and the need for adjunct treatment used synergistically to complement pharmacological management are well established in the scientific literature. The effectiveness of medical hypnosis for reducing the pain and anxiety associated with numerous painful and distressful procedures in adults, including burn wound treatment procedures, is also well documented [7, 33–38, 82]. If our study’s hypothesis holds true, hypnotherapeutic techniques could find a prominent, evidence-based place in the “toolkit” at the pediatric burn care staff’s disposal. They may use this enhanced, sophisticated form of communication to decrease suffering and complement existing pharmacological management of burned children worldwide.

If the intervention is shown to be effective, possible next steps for wider clinical implementation could include training burn care staff to use hypnotic techniques or developing a short film that could be viewed by children in the waiting room prior to scheduled wound care procedures. Creating such a film that could induce (or facilitate) hypnosis for children and invite dissociation from noxious and anxious perceptions is a potential future research direction that could build on this study’s results.

Continuing to identify connections between pain, anxiety, stress, and healing time in acute burn wound treatment is crucial for patients and health care providers and has medical applications and implications beyond the field of burns.

### Trial status

This trial has commenced and recruitment is expected to be completed by the end of 2015.

## Abbreviations

ABMH: American Board of Medical Hypnosis
ASCH: American Society of Clinical Hypnosis
AUS: Australia
CCBTR: Centre for Children’s Burns and Trauma Research
CONSORT: Consolidated Standards of Reporting Trials
CPSS: Child Posttraumatic Stress Disorder Symptom Scale
CPTSD-RI: Child Posttraumatic Stress Disorder Reaction Index
DSM-5: Diagnostic and Statistical Manual of Mental Disorders, Fifth Edition
FLACC: Face, Legs, Activity, Cry, Consolability pain scale
FPS-R: Faces Pain Scale - Revised
*g*: gravitational force
GEE: generalized estimating equation
HR: heart rate
HREC: Human Research Ethics Committee
ITT: intention-to-treat
LCCH: Lady Cilento Children’s Hospital
LDI: laser Doppler imager
MCID: minimum clinically important difference
NRS: numeric rating scale
PLPBC: Pegg Leditschke Paediatric Burns Centre
PTSD: Posttraumatic stress disorder
rCBF: regional cerebral blood flow
RCT: randomized controlled trial
SD: standard deviation
SHCS-C: Stanford Hypnotic Clinical Scale for Children
SPIRIT: Standard Protocol Items: Recommendations for Interventional Trials
SPSS: Statistical Package for the Social Sciences
TBSA: total body surface area
TIDieR: Template for Intervention Description and Replication
VAS: visual analog scale
VAS-A: visual analog scale for anxiety
YCPC: Young Child Posttraumatic Stress Disorder Checklist
